# Short stature: an ordinary sign for an unordinary diagnosis

**DOI:** 10.1186/s13052-017-0381-9

**Published:** 2017-07-28

**Authors:** Paolo Cavarzere, Valentina Bortolotti, Michela Capogna, Margherita Guarnieri, Francesca Lucca, Rossella Gaudino, Stefano Marzini, Claudia Banzato, Franco Antoniazzi

**Affiliations:** 1 Pediatric Clinic, Department of Pediatrics, University Hospital of Verona, Piazzale Stefani 1, 37126 Verona, Italy; 2 Pediatric Unit, Hospital of Feltre, Feltre, Italy; 3 Regional Center for the diagnosis and treatment of children and adolescents rare skeletal disorders, Pediatric Clinic, Department of Surgical Sciences, Dentistry, Gynecology and Pediatrics, University of Verona, Verona, Italy

**Keywords:** Short stature, Shwachmann-Diamond syndrome, Neutropenia, Hypertransaminasemia, Growth retardation

## Abstract

**Background:**

Short stature (SS) is a relatively early sign of poor health. Only in 5% of cases we can explain it through the presence of endocrinological pathologies. Therefore, if SS is present since the first months of life, it is necessary to investigate all systemic disorders with secondary effects on growth.

**Case presentation:**

We report the case of a 16-months-old male infant with severe SS apparently not associated with other clinical signs or symptoms. The patient arrived to our attention after he was hospitalized for an Echovirus enteritis, associated to moderate neutropenia (800/mm^3^) and hypertransaminasemia (AST 116 U/L, ALT 88 U/L) at the age of 13 months. SS was detected in that occasion. Since SS persisted even after the complete resolution of enteritis symptoms, he was taken care by our unit.

**Conclusions:**

SS appeared in the first months of life and associated with moderate neutropenia and hypertransaminasemia led us to the diagnosis of Shwachmann-Diamond syndrome. We recommend paying further attention to this condition during the differential diagnosis of children with severe SS.

## Background

Growth is one of the most fundamental tasks of childhood development and short stature (SS) or growth retardation are regarded as relatively early signs of poor health [1]. Although in presence of SS pediatricians often suggest an endocrinology evaluation, a broad spectrum of diseases other than those due to endocrine causes may be at the origin of this clinical sign. In fact, proportionate SS is due to endocrinopathies only in ∼ 5% of cases. In most cases, systemic disorders with secondary effects on growth should be considered; among them we can remember undernutrition, gastro-intestinal, cardio-pulmonary, renal and rheumatologic diseases, cancer, immunodeficiency, metabolic disorders, and psychosocial deprivation [2]. Consequently, the aim of the endocrinology evaluation for a child with SS is to identify the cause of this condition with particular attention to recognize the presence of undetected diseases.

## Case presentation

**Fig. 1:**
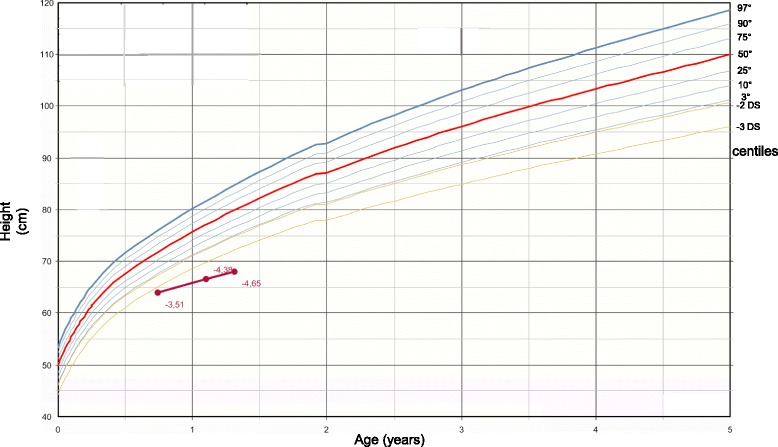
in figure the growth chart of our patients is represented (Who, 2006)

A male infant of 16 months was sent to our Pediatric Endocrinology Centre for growth retardation reported from the third month of life (Fig. 1).

He was born at term by vaginal delivery after an uneventful pregnancy. Birth weight was 2930 g (−1.05 standard deviations [SD]), birth length 49 cm (−0.50 SD). Parents were not related and had a normal stature (target height 174.5 cm). He lived with his parents in a mountain area and had no known allergies, nor contact with infections. No noteworthy disease was reported in family history. Three months before our evaluation, the patient was admitted to another hospital for Echovirus enteritis, which healed normally. SS was detected in that occasion. After the complete resolution of enteritis symptoms, since SS persisted, he underwent different laboratory screening test: blood count, red blood cell indices, leucocyte differentiation, erythrocyte sedimentation rate, serum creatinine, electrolytes, calcium, phosphate, alkalin phosphatase, transaminases, albumin, iron and ferritin. These exams revealed moderate neutropenia (800/mm^3^) and hypertransaminasemia (AST 116 U/L, ALT 88 U/L) which were interpreted as consequence of the enteral infection. Moreover, immunoglobulins, serological tests for celiac disease, ceruloplasmin, anti-nuclear antibody dosage, stool calprotectin, fecal occult blood test, fecal eosinophils, abdominal and heart ultrasonography were performed with normal results.

At our clinical examination, the baby appeared in good conditions, without dysmorphic features and with normal body proportions. While cardiothoracic, abdominal and genital evaluations were normal, a psychomotor delay emerged: he presented a hypotonia of lower limbs and he was not able to walk autonomously. Poor growth was confirmed: bodyweight 7.8 kg (−2.55 SD), height 68 cm (−4.-4.38 SD SD), cranial circumference 46 cm (−1.50 SD), growth velocity 7 cm/year (−2.22 SD).

**Fig. 2:**
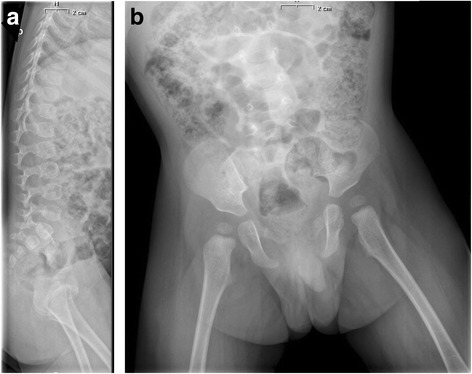
in figure the spine and pelvis X-ray in laterocervical **a** and in anteroposterior **b** projections is represented

We decided to perform further examinations. We identified a slight anemia (Hb 10.1 g/dL) and confirmed a moderate neutropenia (600/mm^3^) and a mild hypertransaminasemia (AST 91 U/L, ALT 102 U/L). Hormonal assays (ACTH, cortisol, GH, TSH, fT4), performed to rule out endocrinological impairment, were normal. We identified only a low value of IGF1 (2.94 nmol/L, −5.8 DS). After finding low fecal elastase, we performed a sweat test; its normal values permitted to exclude the suspect of cystic fibrosis. Celiac disease was also ruled out again. Spine and pelvis X-ray did not show focal lesions, but highlighted coxa valga, ossification delay and oval vertebrae (Fig. 2). Bone age exam showed a severe ossification delay.

In consideration of poor growth, psychomotor delay, anemia, neutropenia, hypertransaminasemia and low fecal elastase, we suspected a Shwachmann-Diamond syndrome (SDS). Consequently, a complete pancreatic assay was performed with the identification of low fecal chymotripsyn and low plasmatic levels of amilase and lipase. Subsequently, genetic analysis for SBDS gene was performed and resulted in compound heterozygosis for c.183-184TA > CT + 258 + 2 T > C, confirming the clinical suspect.

## Discussion and conclusions

The SDS is a rare autosomal recessive disorder, described for the first time in 1964, whose estimated incidence is of 1 in 77,000 people [3]. The SBDS gene, located on long arm of chromosome 7 (7q.11) is mutated in 90% of patients. The protein encoded by SBDS gene appears to be involved in RNA metabolism or ribosome function, affecting exocrine pancreas, bone, cartilage and bone marrow cells development, but its function is still unknown. Consequently, the clinical manifestations are related to exocrine pancreatic insufficiency, with malabsorption and malnutrition; to bone marrow failure, with neutropenia, anemia, thrombocytopenia or pancytopenia; to skeletal anomalies, with abnormal development of growth plates and metaphyses, delayed bone age as well as progressive deformities and pathological fractures. All these symptoms are associated with SS and mild cognitive impairment. Classically SDS presents neutropenia associated with diarrhea [4]; in our case these typical features were misinterpreted as viral enteritis. Furthermore, on this occasion hypertransaminasemia and failure to thrive were also explained as consequence of this acute infection. In fact, it is known that acute viral gastroenteritis may cause elevation of AST and ALT level [5] and are associated with weight loss and growth retardation [6]. Similarly the association between neutropenia and gastroenteritis is known and some authors suggest that mild neutropenia accompanying diarrhea does not require further evaluation, unless it persists or is associated with other factors such as sepsis [7]. Therefore, only the subsequent evaluation after the acute infectious episode suggested a more severe disorder. In particular, three symptoms were decisive for our diagnosis: the presence of neutropenia; the persistent hypertransaminasemia; and finally growth failure.

Since the most common causes of neutropenia are acquired and due to viral infection, drugs and autoimmune etiology [8], the persistence of neutropenia in our patient despite the absence of infection made us assume that it was due to a chronic situation. Moreover, the association between neutropenia and anemia was suggestive of pancytopenia. The pancytopenia with an important neutropenia was the clinical sign that led to the final diagnostic hypothesis. In fact SDS, along with Fanconi syndrome, dyskeratosis congenita and amegakaryocytic thrombocytopenia, is among the main congenital causes of pancytopenia. In patients with SDS the cytopenias are secondary to marrow failure and neutropenia is the most common hematological abnormality, being reported in 88% to 100% of patients. It can be either intermittent or persistent, occurring with recurrent infections. Therefore, the viral enteritis presented by our patient was probably due to his neutropenia. Among other haematological signs, normochromic normocytic anemia with low reticulocytes occurs in up to 80% of SDS patients and it is often asymptomatic [9].

Another hall-mark of SDS is exocrine pancreatic dysfunction with or without nutrient maldigestion. Pancreatic dysfunction is usually diagnosed within the first six months of life and in 90% of patients during the first year. Clinical manifestations range widely from severe dysfunction with significant nutrient malabsorption, steatorrhea, and resultant failure to thrive, to completely asymptomatic. Many patients with SDS spontaneously improve with age; several studies, based on evidence of normal fat absorption in up to 50% of patients, have shown that enzyme supplementation can be stopped by 4 years [10]. The typical clinical findings of exocrine pancreatic dysfunction can be documented with low serum concentrations of the digestive pancreatic enzymes, low levels of fecal elastase and with supportive features such as abnormal fecal fat balance study of a 72-h stool collection, pancreatic lipomatosis at ultrasonography, reduced levels of fat soluble vitamins, reduced pancreatic enzyme secretion following quantitative pancreatic stimulation testing with cholecystokinin and secretin. In our patient, persistent failure to thrive, associated with diarrhea, induced us to measure fecal elastase; its low level enabled us to suspect exocrine pancreatic dysfunction. On the contrary, his abdominal ultrasonography was completely normal and did not highlight pancreatic alterations nor hepatomegaly. Nevertheless his transaminase levels increased over time and were essential for the diagnosis. Hepatomegaly and liver dysfunction with elevated serum aminotransferase concentration, in fact, are seen in up to 75% of patients with SDS, most often in infants and young children, and tend to resolve with age [11].

Although our patient had no dysmorphic features and did not present evident body disproportions, probably also due to his young age, we suspected slight lower limbs alterations. His delay in walking finally suggested us to perform a spine and pelvis X-ray. These exam evidenced a coxa valga, an ossification delay and some oval vertebrae. These alterations, though not specific for SDS, are part of the typical variety of bone anomalies in SDS. Typical feature of SDS is metaphyseal chondrodysplasia. The radiographic findings may be subtle in early childhood such as mild rib shortening and distal rib flaring or cupping, and become more apparent as the child grows, particularly in the lower limbs. The metaphyseal changes lead to asymmetrical metaphyseal growth and deformity. Moreover, generalized disturbance in bone metabolism, as well as delayed appearance of secondary ossification centers, has been shown in SDS; these symptoms cause bone age delay and abnormal bone turnover, with decreased activity of both osteoclasts and osteoblasts [12, 13]. In conformity with these data, the bone age of the patient showed a severe ossification delay.

Growth failure is a common feature of SDS and is described in most cases since the first months of life, even if its causes are not fully understood. It seems only to a small extent caused by skeletal abnormalities, chronic malnutrition, pancreatic insufficiency and recurrent infections with only partial response to pancreatic extracts [14]. An associated growth hormone deficiency (GHD) is rarely reported; in our patient we did not investigated a GHD, but we identified low level of IGF1 that, however, may be also correlated to his malnutrition. It is known that nutrition plays a crucial role in growth during early infancy, and a poor nutritional status may be also a cause of growth retardation [15]. Anyhow, SS and poor growth rate can be the first manifestations of undetected diseases in children. It is indeed estimated that about 20% of children with a height less than 2 SD below the mean and around 50% of the children with a height less than 3 SD below the mean have a pathological reason for their small size [16]. Poor growth can be caused by a great diversity of congenital or acquired conditions, not only endocrinological, such as cystic fibrosis or celiac disease, which, as mentioned above, we have excluded in our patient. In order to recognize these conditions, some laboratory exams are needed, but there is no consensus about which test should be performed [17]. Hematological parameters are suggested by all authors as first-step evaluation, because the association between anemia and SS is a nonspecific marker of a growth related disorder; other tests as transaminases assay are instead under debate. Whereas some authors recommend to investigate the transaminases in presence of SS with unknown etiology [17], other authors do not include this parameter in routine investigations, because they consider that short children with liver disorder are usually not asymptomatic [1, 18]. We identified the cause of our patient’s SS only through the association between growth retardation, neutropenia and elevated transaminases’ levels in absence of specific symptoms. In fact, as above mentioned, persistent hypertransaminasemia is one of clinical signs of SDS [19]. Consequently, we suggest to always evaluate transaminases in children with SS appeared in the first months of life.

In this case, clinical history (growth retardation, psychomotor delay assessment), together with silent familiar history, no-specific physical examination, first level laboratory testing abnormalities (anemia, neutropenia, hypertransaminasemia), led to second level investigations on blood and on fecal samples and subsequently genetic analysis, which confirmed SDS diagnosis. Even if a drawback in our diagnostic process may be the extended variety of tests performed, we believe that they allowed to properly focus on the most specific abnormalities, without invasive methods and with efficient and rapid insight of the diagnosis.

In conclusion, we recommend physicians to routinely measure transaminases levels in infants presenting severe SS in the first months of life. Moreover, when SS is associated with moderate neutropenia and hypertransaminasemia, it is mandatory to hypothesize a SDS and to proceed with further exams, such as the analysis of fecal elastase, a complete pancreatic assay and the genetic of SBDS gene, to confirm the clinical suspect.

## Abbreviations

SD: Standard deviations
SDS: Shwachmann-Diamond syndrome
SS: Short stature
